# Modelling the Gastrointestinal Carriage of Klebsiella pneumoniae Infections

**DOI:** 10.1128/mbio.03121-22

**Published:** 2023-01-04

**Authors:** Ricardo Calderon-Gonzalez, Alix Lee, Guillermo Lopez-Campos, Steven J. Hancock, Joana Sa-Pessoa, Amy Dumigan, Ronan McMullan, Eric L. Campbell, Jose A. Bengoechea

**Affiliations:** a Wellcome-Wolfson Institute for Experimental Medicine, School of Medicine, Dentistry and Biomedical Sciences, Queen's University Belfast, Belfast, United Kingdom; Institut Pasteur

**Keywords:** gut colonization, *Klebsiella pneumoniae*, capsule polysaccharide, type VI secretion system

## Abstract

Klebsiella pneumoniae is a leading cause of nosocomial and community acquired infections, making K. pneumoniae the pathogen that is associated with the second largest number of deaths attributed to any antibiotic resistant infection. K. pneumoniae colonizes the nasopharynx and the gastrointestinal tract in an asymptomatic manner without dissemination to other tissues. Importantly, gastrointestinal colonization is a requisite for infection. Our understanding of K. pneumoniae colonization is still based on interrogating mouse models in which animals are pretreated with antibiotics to disturb the colonization resistance imposed by the gut microbiome. In these models, infections disseminate to other tissues. Here, we report a murine model to allow for the study of the gastrointestinal colonization of K. pneumoniae without tissue dissemination. Hypervirulent and antibiotic resistant strains stably colonize the gastrointestinal tract of in an inbred mouse population without antibiotic treatment. The small intestine is the primary site of colonization and is followed by a transition to the colon over time, without dissemination to other tissues. Our model recapitulates the disease dynamics of the metastatic K. pneumoniae strains that are able to disseminate from the gastrointestinal tract to other sterile sites. Colonization is associated with mild to moderate histopathology, no significant inflammation, and no effect on the richness of the microbiome. Our model sums up the clinical scenario in which antibiotic treatment disturbs the colonization of K. pneumoniae and results in dissemination to other tissues. Finally, we establish that the capsule polysaccharide is necessary for the colonization of the large intestine, whereas the type VI secretion system contributes to colonization across the gastrointestinal tract.

## INTRODUCTION

Klebsiella pneumoniae is one of the pathogens sweeping the world in the antibiotic resistance pandemic. A recent analysis of the global burden of antibiotic resistant infections revealed that more than 250,000 deaths are associated with K. pneumoniae infections (1), making K. pneumoniae the pathogen that is associated with the second largest number of deaths (after Escherichia coli) attributed to any antibiotic resistant infection. In particular, carbapenem-resistant *K. pneumoniae* and third-generation, cephalosporin-resistant K. pneumoniae account for at least 100,000 deaths (1). In addition, Klebsiella species are a known reservoir for antibiotic resistant genes, which can be spread to other Gram-negative bacteria. Not surprisingly, the World Health Organization has singled out K. pneumoniae as an urgent threat to human health.

K. pneumoniae is a member of the human microbiota that is found in the mouth, nares, skin, and gastrointestinal tract. In Western countries, up to 5% of healthy humans from the community are nasopharyngeally colonized with K. pneumoniae, and this percentage may be as high as 30% in Asian countries (2). In healthy individuals in Western countries, the colonization of the gastrointestinal tract ranges from 5% to 35%, and this can reach up to 60 to 70% in Asian countries (2). Importantly, clinical studies demonstrate that gastrointestinal colonization is a requisite for infection (3, 4). This colonization is asymptomatic and, therefore, these healthy subjects could act as silent carriers from which K. pneumoniae may cause disease when the host is compromised or may act as a source of transmission. A point of concern is the colonization by hypervirulent K. pneumoniae (2). These strains are prevalent in Asia, and they have the capacity for metastatic spread. Alarmingly, there are reports of hypervirulent strains becoming multidrug resistant (5–8).

Significant knowledge gaps exist regarding the host factors that influence gastrointestinal colonization, although recent data indicate that age and alcohol consumption are associated with increased colonization (9). In addition, antibiotic treatment seems to predispose individuals to colonization (9) and, in the clinical setting, may result in the dissemination of K. pneumoniae from the gastrointestinal tract to other tissues, thereby resulting in sepsis and other life-threatening complications (3, 4, 10). These observations suggest that the commensal gut microbiota provide a barrier to K. pneumoniae colonization. Indeed, a number of studies in mice demonstrate that antibiotic pretreatment facilitates K. pneumoniae colonization (11).

The vast majority of our knowledge regarding K. pneumoniae-associated gastrointestinal colonization comes from mouse models in which the animals are pretreated with antibiotics (11), thereby resulting in an infection that disseminates to other tissues. These models do not recapitulate the asymptomatic colonization of healthy human subjects harboring an undisturbed microbiome. Furthermore, these models also present limitations in their identification of the K. pneumoniae factors that are implicated in the gut colonization, as the colonization resistance imposed by the microbiome is impaired. Also, there may be changes in the immune response following infection. Undoubtedly, these changes in the gut microenvironment affect the host-K. pneumoniae interface, resulting in changes in the factors deployed by the pathogen to ensure colonization.

Here, we describe a murine model that allows for the study of the gastrointestinal colonization of K. pneumoniae. We demonstrate that K. pneumoniae can stably colonize the gastrointestinal tract of an inbred mouse population without antibiotic pretreatment. We characterize the colonization dynamics by K. pneumoniae and show that antibiotic treatment triggers the dissemination of the infection. Finally, we establish the role of the capsule polysaccharide (CPS) and implicate the type VI secretion system (T6SS) in the colonization of the gastrointestinal tract.

## RESULTS

### Establishing K. pneumoniae colonization of the murine intestinal tract.

We sought to establish a murine model of gastrointestinal tract colonization by K. pneumoniae that would mimic the asymptomatic gastrointestinal colonization of healthy individuals without dissemination to other tissues. Previous studies have used oral gavage and pretreatment with antibiotics to disrupt the colonization resistance imposed by the microbiome (11). We first tested the ability of K. pneumoniae CIP52.145 (here, Kp52145) to colonize the gastrointestinal tracts of adult mice (8 to 9 weeks) of both sexes without antibiotic treatment. This strain belongs to the K. pneumoniae KpI group, and it encodes all virulence functions associated with invasive community-acquired disease in humans (12, 13). A pilot experiment using oral gavage and a dose of 10^8^ CFU per mouse did not result in colonization, as measured by the presence of bacteria in the feces via plating on Simmons citrate agar with inositol (SCAI) medium (14) at 10 days postinfection. Fecal shedding is a widely used substitute for colonization density in the gastrointestinal tract. SCAI medium consists of Simmons citrate agar and inositol. Klebsiella spp. grow as yellow colonies, whereas, apart from a few *Enterobacter* and *Citrobacter* strains, other typical *Enterobactericeae* that are found in the gut grow as blue colonies due to their utilization of citrate (14). We reasoned that the lack of detection of colonization could be the result of low numbers of bacteria reaching the gastrointestinal tract. One of the innate barriers protecting the gastrointestinal tract from infections is the gastric acid of the stomach. Therefore, we hypothesized that colonization would be facilitated if the gastric acid of the stomach was quenched. Indeed, the treatment of mice with sodium bicarbonate 5 min before the oral gavage with K. pneumoniae resulted in gastrointestinal colonization by Kp52145 (Fig. S1A).

10.1128/mbio.03121-22.1 FIG S1 Sodium bicarbonate treatment facilitates K. pneumoniae gut colonization. (A) CFU per gr of small intestine and colon of mice infected with 10^8^ CFU of Kp52145. Mice were pretreated or not with sodium bicarbonate 5 min before the oral gavage of bacteria. 4 to 5 mice per group were infected. (B) Bacterial loads in the colon and feces of mice infected with the indicated bacterial doses. Samples were obtained 12 days postinfection. 4 to 5 mice per group were analyzed. In all panels, each value is presented as the mean ± SD. *, *P* ≤ 0.05; ****, *P* ≤ 0.0001 for the indicated comparisons, which were determined using a Mann-Whitney U test. Download FIG S1, PDF file, 0.5 MB. Copyright © 2023 Calderon-Gonzalez et al. 2023 Calderon-Gonzalez et al. https://creativecommons.org/licenses/by/4.0/ This content is distributed under the terms of the Creative Commons Attribution 4.0 International license.

**FIG 1:**
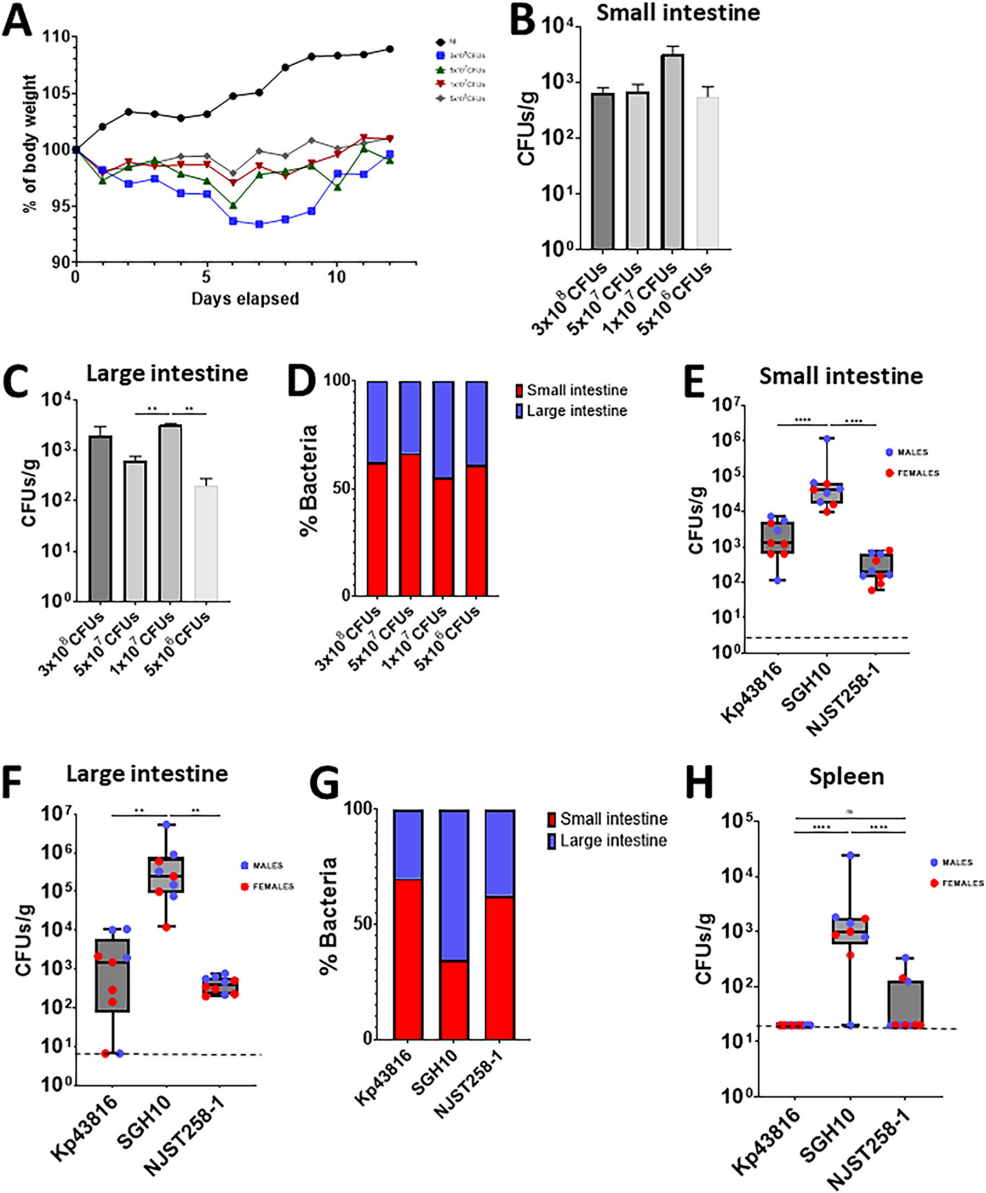
K. pneumoniae colonizes the gut of immunocompetent mice without the need for antibiotic pretreatment. (A) Weight loss of mice infected with different doses of Kp52145 over 12 days. 4 to 6 mice were included in each group, except in the group infected with 3 × 10^8^ CFU, in which three mice died within 5 days postinfection. (B and C) CFU per gr of small (B) and large intestine (C) of the mice infected in panel A at 12 days postinfection. (D) The relative distribution of K. pneumoniae across the intestine sections was determined as 100% of the combined bacterial loads of the small and large intestine sections. (E and F) Bacterial loads in the small (E) and large (F) intestines of mice infected with Kp43816, SGH10, and NJST258-1. In each group, 9 to 10 mice were infected. (G) Relative distribution of K. pneumoniae strains across the intestine sections. (H) Bacterial loads in the spleen of infected mice with different K. pneumoniae strains at 12 days postinfection. Dashed lines indicate the plating detection limit. In all panels, each value is presented as the mean ± SD. **, *P* ≤ 0.01; ****, *P* ≤ 0.0001; ns, *P* > 0.05 for the indicated comparisons, which were determined using a one way-ANOVA with the Bonferroni correction for testing multiple comparisons.

Having established that sodium bicarbonate treatment facilitated colonization, we next sought to determine the minimal dose of K. pneumoniae that would result in a reproducible gastrointestinal colonization. Mice were infected with four bacterial concentrations, and the weight loss, as an indication of health status, was measured daily. 3 of the 6 mice infected with 3 × 10^8^ CFU reached the humane threshold for euthanization within 5 days postinfection. The remaining 3 mice lost 6% of their weight by day 8 postinoculation, although they subsequently recovered weight and ultimately reached their preinoculation weights. Mice infected with 5 × 10^7^, 1 × 10^7^, and 5 × 10^6^ CFU also lost weight, with the weight loss peaking at day 6 postinfection, before the mice starting to regain weight. After, we observed no more than a 5% difference between the infected and noninfected mice (Fig. 1A). At the end of the experiment (day 12 postinfection), the mice were euthanized, and the bacterial loads in the intestine were quantified via plating. Kp52145 was detected in the small and large intestines (Fig. 1B and C). No significant differences between doses were found in the small intestine (Fig. 1B). In contrast, the mice infected with 1 × 10^7^ had higher bacterial loads in the large intestine than did those infected with either 5 × 10^7^ or 5 × 10^6^ (Fig. 1C). Next, we determined the distribution of bacteria along the gastrointestinal tract as a percentage. Our results showed that approximately 60% of bacteria were found in the small intestine, and this was without significant differences between doses (Fig. 1D). Interestingly, the bacterial loads in the feces were at least 10 times lower than those found by plating the tissue (Fig. S1B), suggesting that fecal shedding is not a reliable surrogate of gastrointestinal gut colonization in our model. Taken together, our data suggest that quenching the gastric acid facilitates the colonization of the gastrointestinal tract by K. pneumoniae without the need for antibiotic pretreatment. We chose 1 × 10^7^ as the dose for subsequent experiments, considering its minimal effect on the health of the animals and the levels of bacterial loads across the tissues.

A feature of the K. pneumoniae population structure is its genetic diversity between strains (12). Therefore, we tested the ability of a set of genetically diverse isolates to colonize the gastrointestinal tract. We chose strain ATCC 43816 (here, Kp43816), a widely used isolate in the investigation of K. pneumoniae virulence (15), NJST258-1, an isolate of the carbapenem resistant ST258 epidemic clonal group (16), and SGH10, the reference strain of the hypervirulent CG23 clonal group that is associated with liver abscess infections (17, 18). The three strains colonized the small and large intestine at 12 days postinoculation, although the bacterial loads of strain SGH10 were higher than those of the two other strains (Fig. 1E and F). Similar to that of Kp52145, the distribution of Kp43816 and NJST258-1 within the gastrointestinal tract was significantly higher in the small intestine than in the large intestine (Fig. 1G). No significant differences between Kp43816 and NJST258-1 were observed. In contrast, the distribution of SGH10 was higher in the large intestine than in the small intestine (Fig. 1G) (**, *P* ≤ 0.01, Mann-Whitney U test). Only in mice infected with SGH10 did we observe dissemination to the spleen (Fig. 1H). Collectively, our results suggest that gastrointestinal colonization is a general feature of K. pneumoniae. Furthermore, our results indicate that our colonization model may recapitulate the disease dynamics of metastatic K. pneumoniae strains, such as SGH10, that disseminate from the gastrointestinal tract to other sterile sites.

### Gastrointestinal dynamics of K. pneumoniae colonization.

**FIG 2:**
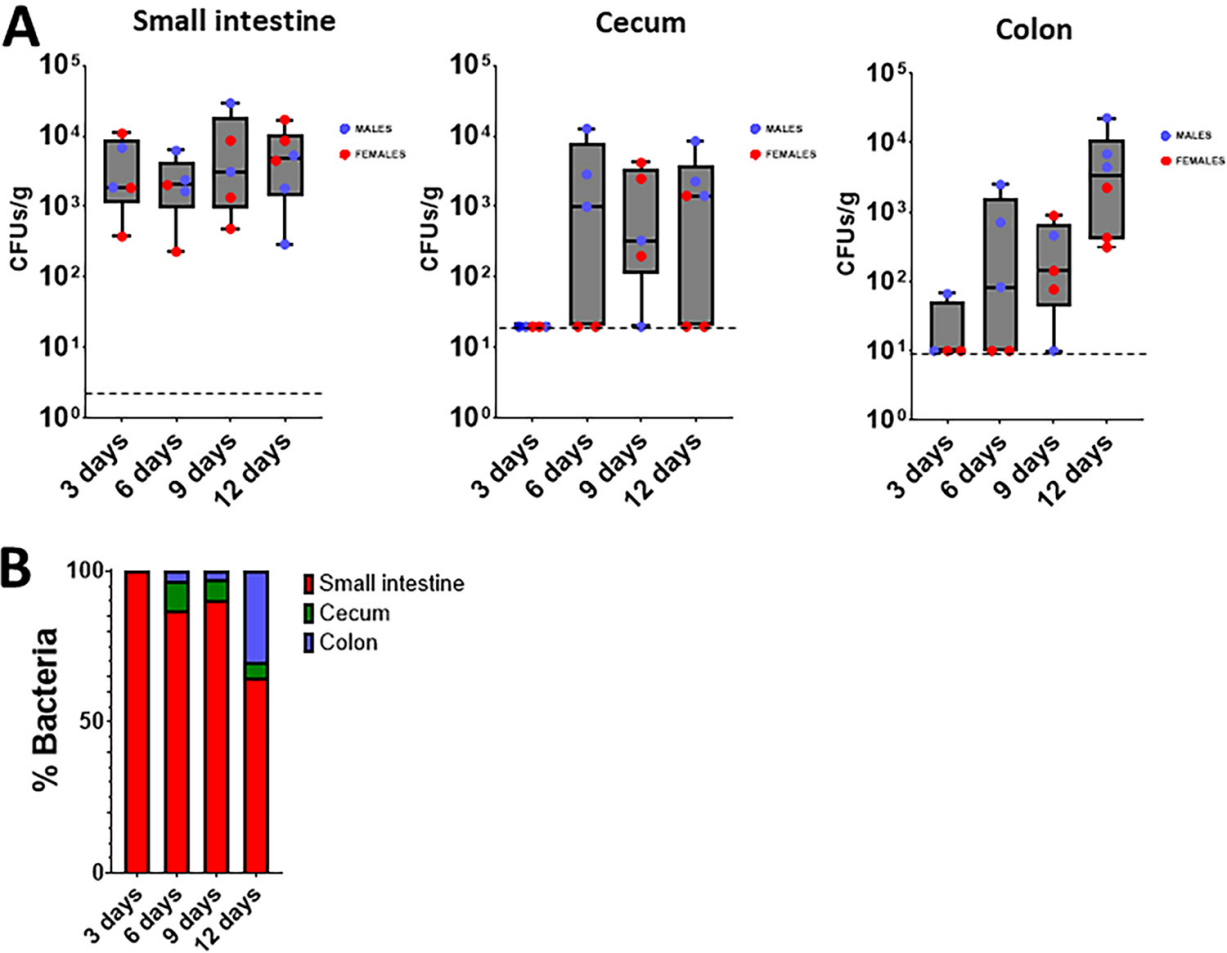
Dynamics of K. pneumoniae gut colonization. (A) Bacterial loads in the different intestine sections of mice infected with Kp52145 on different days postinfection. Each value is presented as the mean ± SD. (B) Relative distribution of Kp52145 across the intestine sections. Dashed lines indicate the plating detection limit.

The differences in bacterial numbers between the small and large intestine led us to explore the colonization dynamics over time in the different sections of the gastrointestinal tract. At 3 days postinfection, bacteria were only detected in the small intestine, not in the cecum or colon (Fig. 2A). The bacterial loads in the small intestine remained consistent until the end of the experiment at day 12 (Fig. 2A). Colonization of the cecum was first detected at 6 days postinfection, and it remained constant until the end of the experiment (Fig. 2A). However, the bacterial loads in the cecum did not reach the levels observed in the small intestine (Fig. 2A). The colonization of the colon was apparent at day 6 postinfection, and it did reach the levels observed in the small intestine at day 12 (Fig. 2A). Distribution analyses of the percentages of bacteria in the three sections of the gastrointestinal tract over time further showed the initial colonization of the small intestine with a progressive increase in the number of bacteria colonizing the colon (Fig. 2B). The dissemination to other tissues, such as the spleen and lung, was not observed at any time point. Together, these results suggest that the primary site of colonization of the gastrointestinal tract by K. pneumoniae is the small intestine and that this is followed by a transition to the colon over time, without dissemination to other tissues.

**FIG 3:**
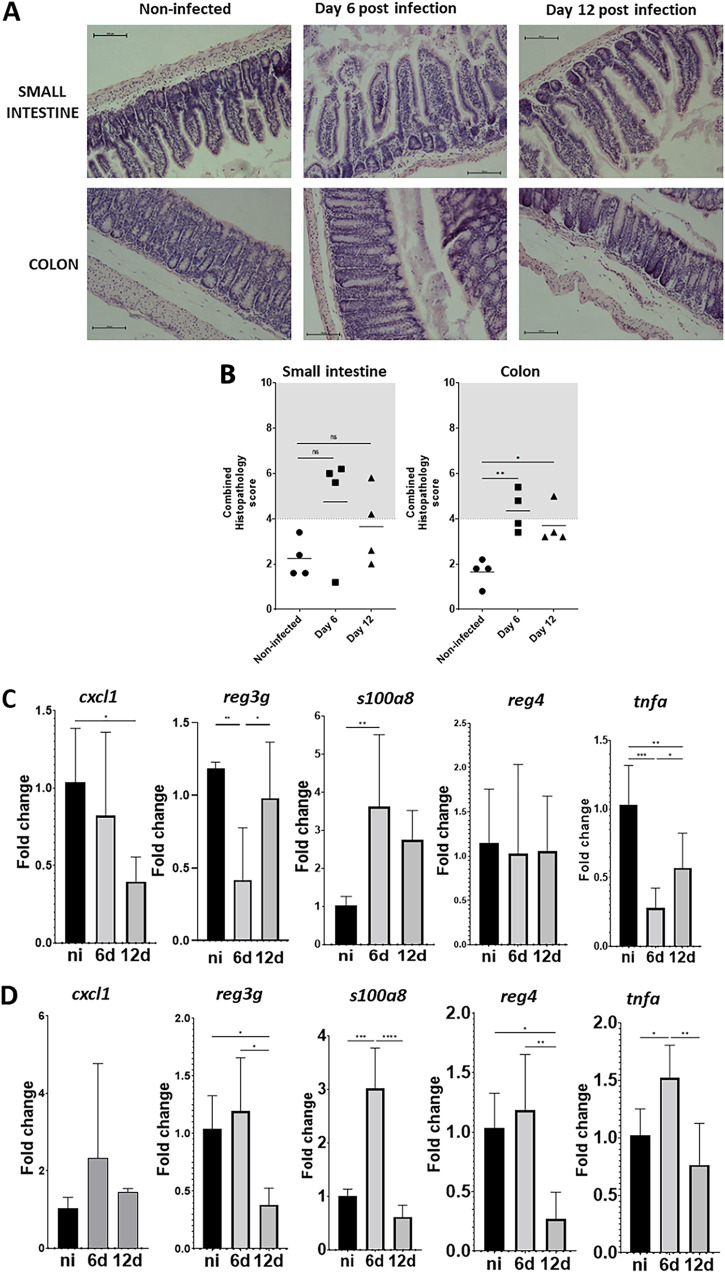
K. pneumoniae colonization does not produce severe tissue damage. (A) Hematoxylin-eosin staining of tissue at different days postinfection with Kp52145. (B) Quantification of the histopathology changes upon infection. Each dot represents a different mouse. Five slides per section of tissue, per time point and per mouse, were scored. (C and D) The *cxcl1*, *reg3g*, *s100o8*, *reg4*, and *tnfa* mRNA levels were assessed by qPCR in the small intestine (C) and in the colon (D) of noninfected mice (black bars) and infected mice (gray bars) at 6 (6 d) and 12 (12 d) days. 5 to 7 mice were analyzed in each group. In panel A, the images are representative of four infected mice. Each value is presented as the mean ± SD. *, *P* ≤ 0.05; **, *P* ≤ 0.01; ***, *P* ≤ 0.001; ****, *P* ≤ 0.0001 for the indicated comparisons, which were determined using a one way-ANOVA with the Bonferroni correction for testing multiple comparisons. No other comparison is significant (*P* > 0.05).

Next, we investigated whether K. pneumoniae colonization is associated with histopathological changes in the gastrointestinal tract. We screened sections for the infiltration of inflammatory cells, the presence of submucosal edema, epithelial damage, or the presence of exudate in the small intestine and colon sections stained with hematoxylin-eosin, according to the criteria shown in Table S1 (19). A combined score between 4 and 6 is considered to be moderate inflammation, whereas a score higher than 7 indicates severe inflammation (19). Fig. 3 shows mild to moderate epithelial damage, the accumulation of exudate, and the infiltration of inflammatory cells at days 6 and 12 postinfection in the small intestines of the infected mice. However, these changes were not significantly different, compared to noninfected mice. Similar mild to moderate changes were observed in the colons of the infected mice at 6 and 12 days postinfection (Fig. 3A and B).

10.1128/mbio.03121-22.6 TABLE S1 Histopathology scoring for murine intestine. Download Table S1, PDF file, 0.01 MB. Copyright © 2023 Calderon-Gonzalez et al. 2023 Calderon-Gonzalez et al. https://creativecommons.org/licenses/by/4.0/ This content is distributed under the terms of the Creative Commons Attribution 4.0 International license.

To provide further evidence that the colonization is not associated with major alterations in the tissue, we next assessed the expression of inflammatory genes associated with the host defense against gut pathogens and inflammation. We detected the upregulation of *s100a8* only at 6 days postinfection in the small intestine and in the colon (Fig. 3C and D). S100a8 is present in intestinal tissue during inflammation, and it is considered to be a valuable biomarker of colonic inflammatory conditions (20). Kp52145 colonization did not upregulate the inflammatory marker *cxcl1* in either the small intestine or the colon (Fig. 3C and D), whereas the infection upregulated *tnfa* only at 6 days postinfection in the colon (Fig. 3C and D). The infection did not induce the expression of the regenerating, islet-derived family members 4 and 3g (Fig. 3C and D). The upregulation of Reg proteins was observed in intestinal inflammation, and some of the Reg family members show antimicrobial activity (21, 22). These results indicate that K. pneumoniae gut colonization does not induce inflammation.

**FIG 4:**
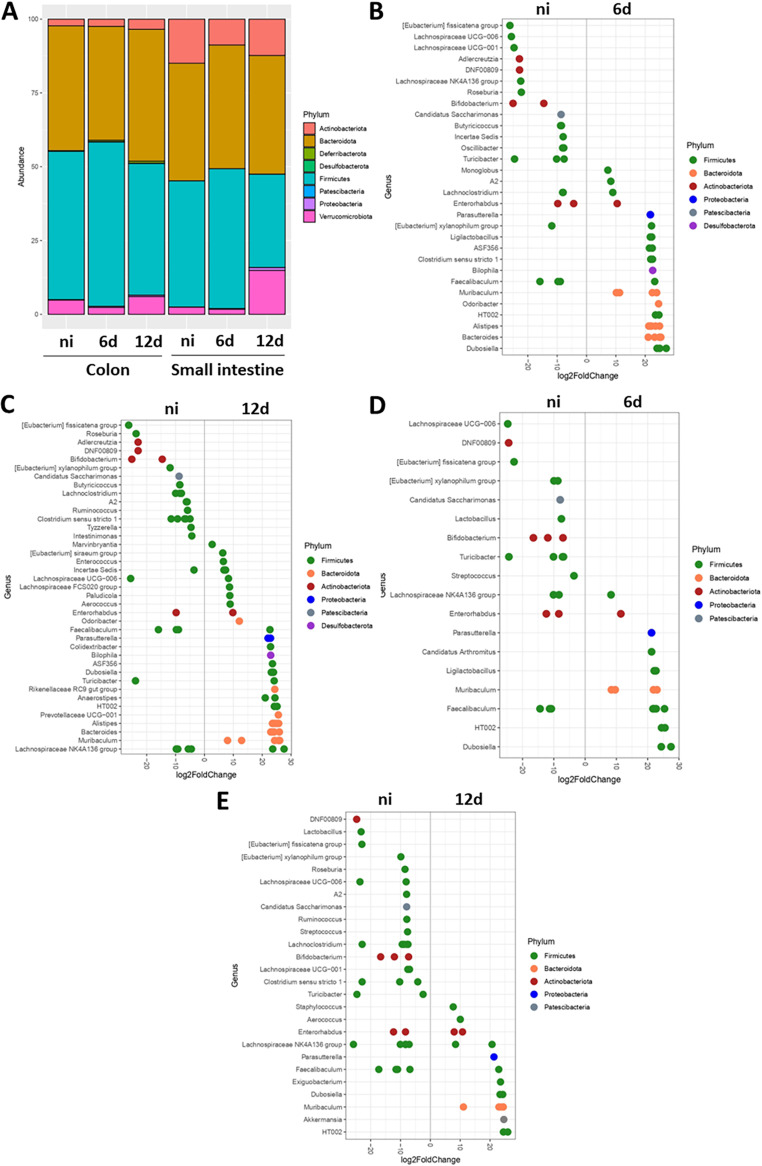
K. pneumoniae colonization has a minor effect on the bacterial gut microbiome. (A) The phyla present in the colon and small intestine of infected mice with Kp52145 were determined by 16S rRNA sequencing. (B and C) Log_2_-fold change of the genera present in the colon of the infected mice at 6 (B) and 12 (C) days postinfection versus the noninfected (ni) mice. (D and E) Log_2_-fold change of the genera present in the small intestine of the infected mice at 6 (D) and 12 (E) days postinfection versus the noninfected (ni) mice. In all panels, 5 to 7 mice were analyzed in each group.

Finally, we sought to determine whether K. pneumoniae colonization perturbs the gastrointestinal tract microbiota. Samples were obtained at 6 and 12 days postinfection from the small intestine and the colon, and the intestinal microbiota were assessed via 16S rRNA sequencing. Control experiments revealed the presence of Kp52145 in the colons of the infected mice (Fig. S2). Kp52145 was detected by a qPCR method that enabled the specific detection of K. pneumoniae by amplifying the intergenic region between the *zur* and *khe* genes, named ZKIR for the *zur-khe* intergenic region (23, 24). No significant differences were observed in the alpha diversity, independent of the test used to measure it (Fig. S3). At the phylum level, colonization with Kp52145 did not lead to dramatic changes in the relative abundance (Fig. 4A). We did not detect K. pneumoniae 16S rRNA gene sequences in these samples, suggesting that K. pneumoniae comprises only a minor component of the host intestinal microbiota.

10.1128/mbio.03121-22.2 FIG S2 Detection of K. pneumoniae by ZKIR-qPCR in colon samples. K. pneumoniae was detected in the colon samples of noninfected (NI) mice and of infected mice at 6 (6 d) and 12 (12 d) days postinfection by the ZKIR-qPCR method. 6 to 7 mice were analyzed in each group. Download FIG S2, PDF file, 0.2 MB. Copyright © 2023 Calderon-Gonzalez et al. 2023 Calderon-Gonzalez et al. https://creativecommons.org/licenses/by/4.0/ This content is distributed under the terms of the Creative Commons Attribution 4.0 International license.

10.1128/mbio.03121-22.3 FIG S3 K. pneumoniae gut colonization does not affect the richness of the microbiome. The diversities of the fecal microbiota of noninfected (ni) mice and of infected mice at 6 (6 d) and 12 (12 d) days postinfection are summarized by 4 indexes. 6 to 7 mice were analyzed in each group. No statistical differences were found. Download FIG S3, PDF file, 0.5 MB. Copyright © 2023 Calderon-Gonzalez et al. 2023 Calderon-Gonzalez et al. https://creativecommons.org/licenses/by/4.0/ This content is distributed under the terms of the Creative Commons Attribution 4.0 International license.

The colon samples were characterized by the abundance of *Firmicutes* and *Bacteriodes*, and the colonization was associated with a relative increase in the abundance of *Desufolbacteria* and *Proteobacteria* at 12 days postinfection. At the genus level, the colonization of the colon at 6 days postinfection resulted in changes within the species of *Actinobacteria*, with a particularly noteworthy decrease in the relative abundance of *Bifidobacterium* and *Adlercreutzia* (Fig. 4B). We also detected changes within the species of *Firmicutes*, namely, a notable increase in the relative abundance of *Clostridium* (Fig. 4B). The *Proteobacteria Parasutterela*, the *Desulfobacterium Biolophila*, and the *Bacteroides Muribaculum*, *Prevotella*, *Alistipes*, and *Bacteroides* were characteristic of a Kp52145-colonized colon (Fig. 4B). Similar findings were obtained when analyzing the samples from 12 days postinfection, except for the relative increase in *Clostridium* (Fig. 4C). The main phyla comprising the small intestine samples were *Actinobacteria*, *Bacteroides*, and *Firmicutes* (Fig. 4A). Only at 12 days postinfection did we detecte changes in the microbiota of the small intestine, with a relative increase in *Verrrucomicrobiota* and *Proteobacteria* and a relative decrease in *Firmicutes* being observed (Fig. 4A). At the genus level, at 6 days postinfection, the conspicuous changes were a decrease in the relative abundance of *Bifidobacterium*, and an increase in the abundance of *Parasutterela* and *Muribaculum* (Fig. 4D). Similar changes were observed at 12 days postinfection (Fig. 4E). Additionally, we observed an increase in the relative abundance of *Akkermansia* and a shift in the genera of *Firmicutes* in the Kp52145-colonized samples. (Fig. 4E). Taken together, these findings demonstrate that although K. pneumoniae colonization did not affect the richness of the microbiome, changes were observed at the genus level. K. pneumoniae colonization results in shifts in the species of *Firmicutes*, a decrease in the relative abundance of the *Actinobacteria Bifidobacterium*, and an increase in the abundance of the *Proteobacteria Parasutterela* as well as species of *Bacteroides.*

### Antibiotic treatment triggers the dissemination of K. pneumoniae from the gastrointestinal tract.

**FIG 5:**
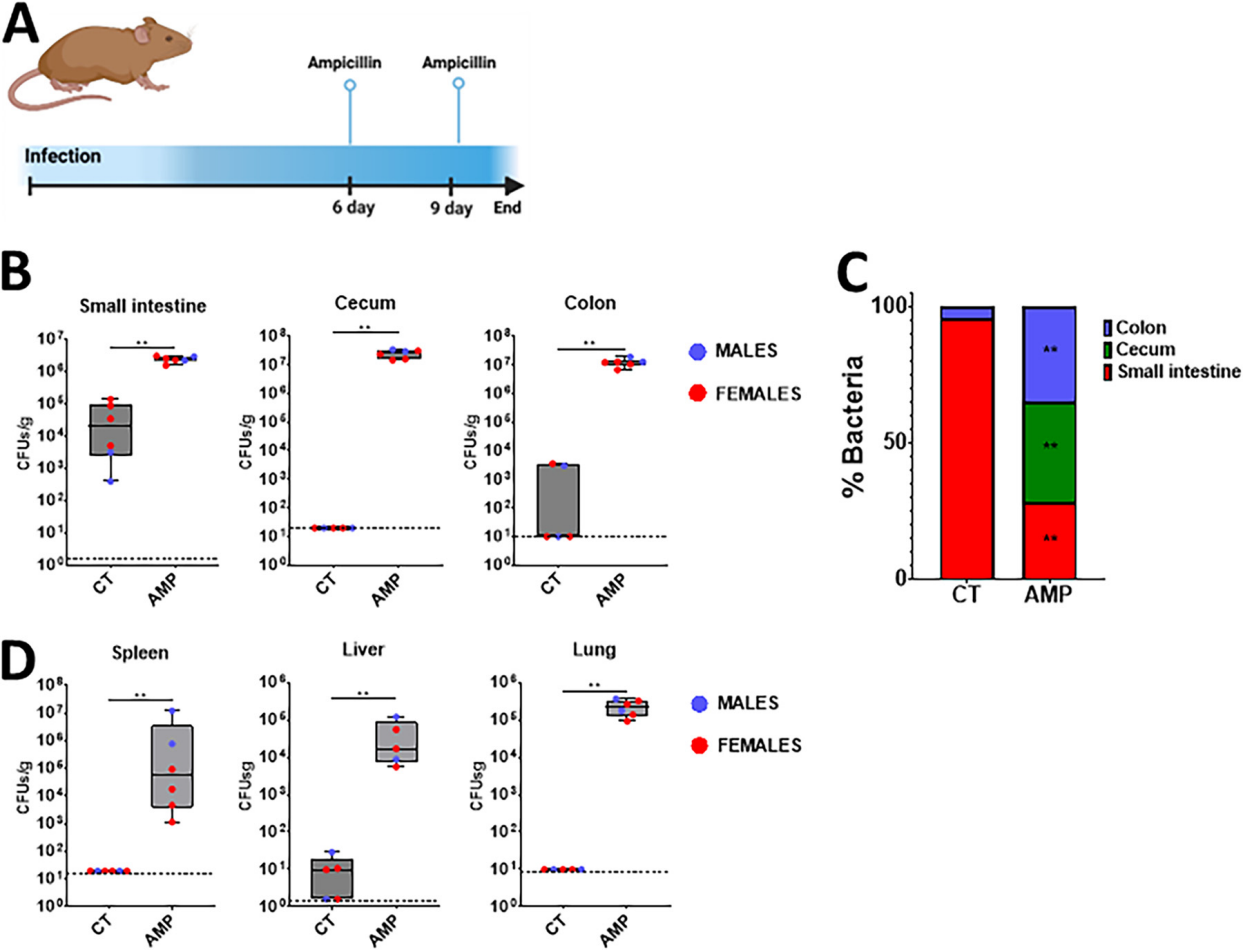
Antibiotic treatment triggers the dissemination of K. pneumoniae from the gastrointestinal tract. (A) Six days after mice were colonized with strain Kp43816, mice were given two doses of ampicillin (i.p.) and 24 h after the last dose mice were euthanized and the bacterial loads were determined. (B) CFU per gr of small intestine, cecum, and colon of mice colonized with Kp43816 and treated with either vehicle solution (CT) or ampicillin (AMP). (C) Relative distribution of Kp43816 across the intestine sections of mice treated with either vehicle solution (CT) or ampicillin (AMP). (D) Bacterial loads in the spleen, liver, and lung of mice colonized with Kp43816 and treated with either vehicle solution (CT) or ampicillin (AMP). Dashed lines indicate the plating detection limit. In all panels, 6 mice were included in each group. Values are presented as the mean ± SD. **, *P* ≤ 0.01; ****, *P* ≤ 0.0001 for the indicated comparisons, which were determined using a Mann-Whitney U test. In panel C, **, *P* ≤ 0.01 for the comparisons, which were determined using a Mann-Whitney U test, of the percentage of bacteria in each of the tissue sections between mice treated with either vehicle solution (CT) or ampicillin (AMP).

In a clinical setting, patients received antibiotic treatment, and evidence indicates that this may result in K. pneumoniae dissemination from the gut to other tissues (10). Therefore, we sought to establish whether antibiotic treatment fosters the dissemination of the K. pneumoniae that colonizes the gastrointestinal tract. Mice were colonized with the ampicillin resistant strain Kp43816, and after 6 days, they received 2 doses of ampicillin (Fig. 5A). Antibiotic treatment resulted in a two log increase in the number of Kp43816 in the small intestine (Fig. 5B). Antibiotic treatment also dramatically increased the levels of Kp43816 in the cecum and colon (Fig. 5B). A distribution analysis further confirmed that antibiotic treatment shifted the colonization of K. pneumoniae from the small to the large intestine (Fig. 5C). Antibiotic treatment also caused the dissemination of Kp43816 from the gut to the spleen, liver, and lungs (Fig. 5D). Taken together, these findings demonstrate that our gut colonization model recapitulates the clinical scenario in which antibiotic treatment disturbs the colonization of K. pneumoniae and results in dissemination to other tissues.

### The capsule and the type VI secretion system contribute to gastrointestinal tract colonization.

**FIG 6:**
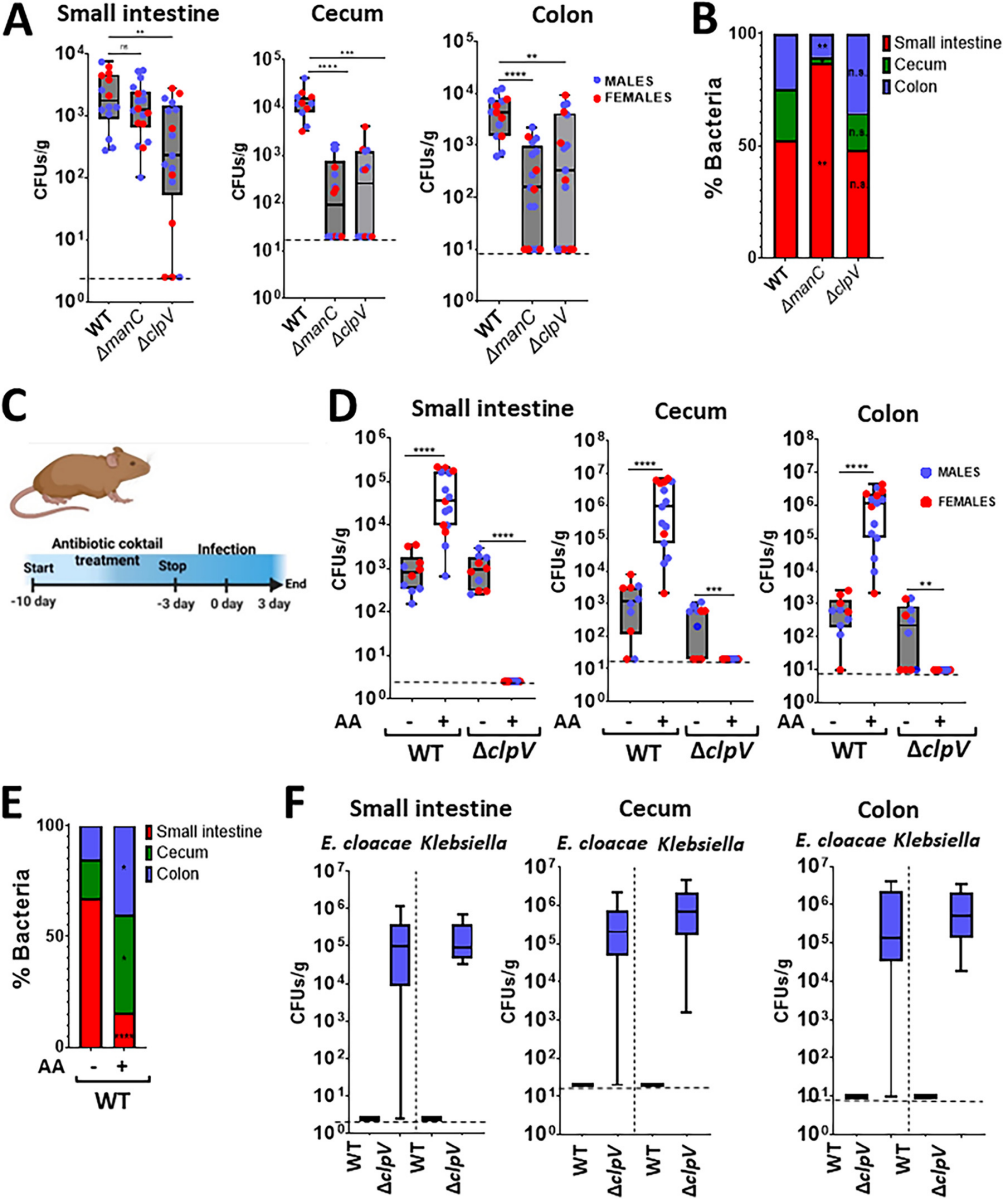
K. pneumoniae CPS and the T6SS are required for gut colonization. (A) CFU per gr of small intestine, cecum, and colon of mice infected with Kp52145, the isogenic *cps* mutant (Δ*manC*), and the T6SS mutant (Δ*clpV*).14 to 17 mice were included in each group in two independent experiments. (B) Relative distribution of Kp52145 and the isogenic *cps* (Δ*manC*) and T6SS (Δ*clpV*) mutants across the intestine sections. (C) To determine the effect of the gut microbiome on the colonization by the T6SS mutant, mice were pretreated with an antibiotic cocktail that was stopped 3 days preinfection. Three days postinfection, the bacterial burdens in tissues were determined. (D) CFU per gr of small intestine, cecum, and colon of mice infected with Kp52145 and the T6SS mutant (Δ*clpV*) that were pretreated or not with the antibiotic cocktail (AA). 10 to 15 mice were included in each group in two independent experiments. (E) Relative distribution of Kp52145 across the intestine sections of mice pretreated or not with the antibiotic cocktail (AA). (F) CFU per gr of small intestine, cecum, and colon of E. cloacae and Klebsiella *spp* that were only found in mice infected with the T6SS mutant (Δ*clpV*) and pretreated with the antibiotic cocktail. In all panels, each value is presented as the mean ± SD. *, *P* ≤ 0.05; **, *P* ≤ 0.01; ***, *P* ≤ 0.001; ****, *P* ≤ 0.0001; ns, *P* > 0.05 for the indicated comparisons, which were determined using a one way-ANOVA with the Bonferroni correction for testing multiple comparisons. In panel B, *, *P* ≤ 0.05; **, *P* ≤ 0.01; ns, *P* > 0.05 for the comparisons, which were determined using a one way-ANOVA with the Bonferroni correction for testing multiple comparisons, of the percentage of bacteria in each of the tissue sections between the mice infected with Kp52145 and each of the mutants. In panel C, *, *P* ≤ 0.05; ****, *P* ≤ 0.0001 for the comparisons, which were determined using a Mann-Whitney U test, of the percentage of bacteria in each of the tissue sections between the mice infected with Kp52145 and those treated or not with the antibiotic cocktail (AA).

The fact that the capsule polysaccharide (CPS) plays a crucial role in the host-K. pneumoniae interface led us to examine the contribution of the CPS to gastrointestinal colonization. There are reports showing that the CPS is necessary for the gut colonization of antibiotic pretreated mice (25, 26). In our model, the *cps* mutant colonized the small intestine, as did the wild-type strain (Fig. 6A). In contrast, the mutant poorly colonized the cecum and colon, suggesting that the CPS is necessary for the colonization of the large intestine. Further confirming this finding, a distribution analysis of the bacteria showed that the levels of the *cps* mutant were higher in the small intestine than in the large intestine (Fig. 6B).

We have recently demonstrated that K. pneumoniae exploits a T6SS for bacteria and fungi antagonism (27). Given the barrier imposed by the gut microbiome to K. pneumoniae gut colonization, we sought to establish whether the T6SS is needed for the colonization of the gastrointestinal tract. Mice were infected with the *clpV* mutant, and the bacterial loads in the different sections of the gastrointestinal tract were determined. ClpV is the AAA^+^ ATPase of the T6SS (28, 29), and in the *clpV* mutant background, K. pneumoniae T6SS is not functional (27). The loads of the *clpV* mutant in the small intestine, cecum, and colon were significantly lower than those of the wild-type strain (Fig. 4A), illustrating the need for a functional T6SS to colonize the gastrointestinal tract by K. pneumoniae. Interestingly, a distribution analysis of the *clpV* mutant did not reveal any difference, compared to the wild-type strain (Fig. 6B), suggesting that the lack of function of the T6SS did not affect the distribution of the mutant across the gastrointestinal tract.

**FIG 7:**
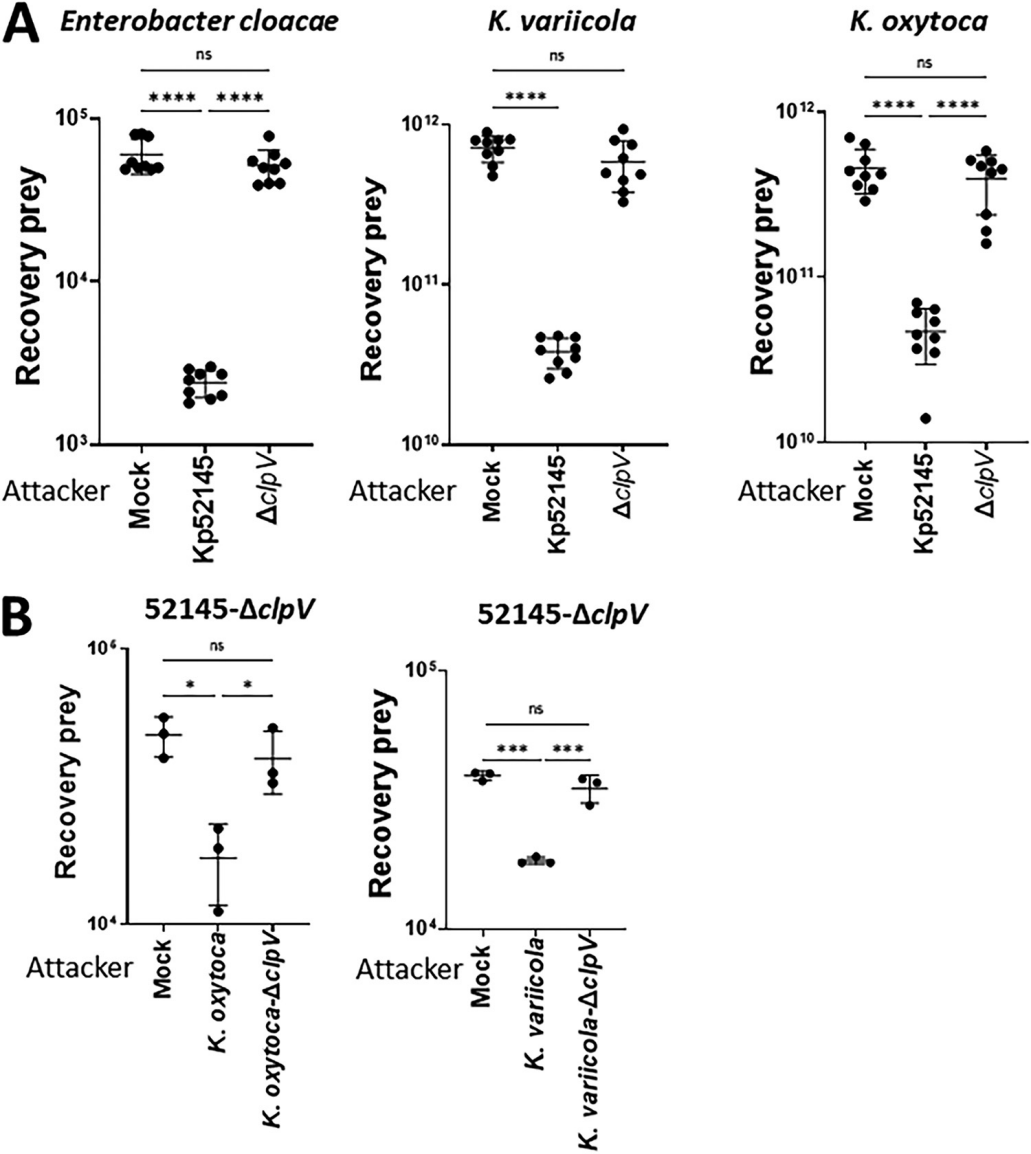
K. pneumoniae antagonizes other *Enterobacteriaceae* of the gut microbiome in a T6SS-dependent manner. (A) Bacterial killing mediated by Kp52145 and the T6SS mutant 52145-ΔclpV (Δ*clpV*) against E. cloacae, K. oxytoca, and K. variicola. Mock, PBS-treated prey. The number of recovered target cells following 6 h of incubation in LB is indicated. (B) Bacterial killing mediated by K. oxytoca and its isogeneic *clpV* mutant (*K. oxytoca*-Δ*clpV*), and by *K. variicola* and its *clpV* mutant (strain *K. variicola*-Δ*clpV*) against 52145-Δ*clpV*. Mock, PBS-treated prey. The number of recovered target cells following 6 h of incubation in LB is indicated. In all panels, each value is presented as the mean ± SD (*n* = 3). *, *P* ≤ 0.05; ***, *P* ≤ 0.001; ****, *P* ≤ 0.0001; ns, *P* > 0.05 for the indicated comparisons, which were determined using a one way-ANOVA with the Bonferroni correction for testing multiple comparisons.

Next, we sought to ascertain whether a reduction in the gut microbiome before infection would mitigate the absence of a functional T6SS due to the limited competition exerted by the gut microbiome. Mice were pretreated with an antibiotic cocktail for 10 days before infection, and after 3 days without treatment, they were infected with the wild-type strain and the *clpV* mutant (Fig. 6C). As we anticipated, there was a significant increase in the Kp52145 loads in the small intestine, cecum, and colon in the mice that were pretreated with antibiotics, compared to the control mice (Fig. 6D). A distribution analysis of the Kp52145 distribution across the gastrointestinal tract showed a shift toward the colonization of the large intestine (Fig. 6E). Only in the antibiotic pretreated mice did we observe the dissemination of Kp52145 to the lungs and spleen (Fig. S4). In contrast, the *clpV* mutant did not colonize the gastrointestinal tract of the mice pretreated with antibiotics (Fig. 6D). This result may indicate that the T6SS is crucial for the colonization of the gastrointestinal tract, even when the colonization resistance imposed by the microbiota is disrupted. However, we noted that in the plates from the pretreated mice that were infected with the *clpV* mutant, colonies that did not metabolize inositol appeared, and other small colonies were inositol positive. A MALDI-TOF analysis revealed that the inositol negative bacteria were classified as Enterobacter cloacae (experiment 1 and 2), whereas the inositol positive bacteria were *K. variicola* (experiment 1) and K. oxytoca (experiment 2). These bacteria colonized in high levels only the small intestine, cecum, and colon of the antibiotic pretreated mice infected with the *clpV* mutant (Fig. 6F). We reasoned that the antibiotic pretreatment triggered a bloom of these bacteria, which were present in low levels in the gut microbiome; however, Kp52145 outcompeted them in a T6SS-dependent manner. To confirm this, we tested whether Kp52145 exerts antibacterial activity against these bacteria in a T6SS-dependent manner. Indeed, quantitative competition assays revealed that Kp52145 killed the E. cloacae and Klebsiella *spp* strains (Fig. 7A). This was not the case when the prey strains were coincubated with the *clpV* mutant (Fig. 7A). To determine whether the Klebsiella *spp* strains outcompeted the *clpV* mutant in a T6SS-dependent manner, we constructed K. oxytoca and *K. variicola clpV* mutants. When the Kp52145 *clpV* mutant was coincubated with any of the T6SS mutants, there was no reduction in the recovery of the prey (Fig. 7B).

10.1128/mbio.03121-22.4 FIG S4 K. pneumoniae disseminates from the gut to other tissues in mice pretreated with the antibiotic cocktail. CFU per gr of lung and spleen of mice infected with Kp52145 that were pretreated or not with the antibiotic cocktail (AA). 10 to 16 mice were included in each group in two independent experiments. Dashed lines indicate the limit of detection. In all panels, each value is presented as the mean ± SD. **, *P* ≤ 0.01; ****, *P* ≤ 0.0001 for the indicated comparisons, which were determined using a Mann-Whitney U test. Download FIG S4, PDF file, 0.5 MB. Copyright © 2023 Calderon-Gonzalez et al. 2023 Calderon-Gonzalez et al. https://creativecommons.org/licenses/by/4.0/ This content is distributed under the terms of the Creative Commons Attribution 4.0 International license.

Taken together, our data demonstrate that the CPS is only necessary for the colonization of the large intestine, whereas the T6SS contributes to colonization across the gastrointestinal tract. Our findings are consistent with the notion that K. pneumoniae exploits its T6SS to compete with the gut microbiome.

## DISCUSSION

In this work, we present a new model of gut colonization by K. pneumoniae upon oral gavage that recapitulates key features of the asymptomatic human gastrointestinal tract colonization by this pathogen. In our model, there is no need to disturb the microbiota of immunocompetent mice to achieve stable colonization, and there is no dissemination to other tissues. The fact that limited weight loss, histopathology, and inflammation were observed during colonization supports the notion that we are modeling asymptomatic colonization without overt infection or disease. Importantly, our model also discriminates between the Klebsiella strains with metastatic capacity and those without. We demonstrate that the small intestine is the primary site of colonization by K. pneumoniae. Finally, we experimentally validate the clinical observation that antibiotic treatment favors K. pneumoniae dissemination from the gut to other tissues.

Previous studies used antibiotic treatment to disrupt the colonization barrier imposed by the gut microbiome to enable K. pneumoniae colonization (11). As our work illustrates, this treatment shifts the distribution of K. pneumoniae within the gut, thereby increasing the colonization of the large intestine and facilitating dissemination to other tissues. Therefore, studies using antibiotic pretreatment recapitulate an invasive gut infection rather than gut colonization. Recently, an oral mouse model of infection also showed the ability of K. pneumoniae to colonize the gut without the need for antibiotic pretreatment (30). In contrast to our model, this model relies on the constant shedding of bacteria from the oropharynx to generate a continuous infection state of the mice, as exemplified by the high fecal shedding of bacteria (30). Although undoubtedly useful, this model does not recapitulate the asymptomatic gut colonization without upper airway infection characteristic of healthy human subjects.

Our findings establish that the primary site of colonization by K. pneumoniae is the mouse small intestine, and there is no dissemination to other tissues. The human small intestine microbiome remains poorly characterized due to the fact this compartment is not easily accessible for sampling in humans. Some data exists, relating to patients undergoing surgery, extensive peroral/nasal intubation, or purging prior to colonoscopy. Only recently, and by sampling the ileostomies of patients who were cured of colorectal cancer, is there data regarding the human small intestine microbiome (31). Supporting the results obtained with our mouse model, K. pneumoniae was found predominantly in the small intestine, compared to the colon.

The microenvironment of the small intestine is remarkably different from that of the large intestine, being comparatively more aerobic than are other sections of the gastrointestinal tract, displaying a lower density of microbiota than do other sections, and displaying a higher concentration of antimicrobial agents than do other sections (32). These peptides create a harsh environment, limiting colonization by enteric pathogens (33). However, work from our laboratory has uncovered a number of Klebsiella factors that mediate antimicrobial peptide resistance (34–40), undoubtedly allowing Klebsiella to survive in this antimicrobial peptide-rich microenvironment. The subsequent colonization of the colon after 6 days, although at lower levels than were observed in the small intestine, suggests that the resistance of the colon against K. pneumoniae colonization is higher than that of the small intestine. Although many factors may contribute to this barrier, our data indicate that the colon microbiome is a crucial one because its reduction dramatically increased the bacterial loads of K. pneumoniae. Notably, this effect was not that marked for the small intestine, suggesting that the microbiome of the small intestine confers limited resistance to K. pneumoniae colonization.

Unlike other *Enterobactericeae*, such as *Salmonella* Typhimurium, Escherichia coli, Citrobacter rodentium, and Vibrio cholerae (41–47), K. pneumoniae colonization was not associated with histopathology changes or with an acute inflammatory response in either the small or the large intestine. This finding does not contradict the clinical reports showing that K. pneumoniae is one of the pathogens associated with inflammatory bowel disease (48). This inflammatory condition triggers a strong dysbiosis, which in turn favors the bloom of pathogens, such as Klebsiella, that are already present within the gastrointestinal tract. Collectively, our results support the notion that the K. pneumoniae colonization of the gastrointestinal tract should be considered an example of stealth behavior. Emerging evidence also indicates that K. pneumoniae stealth strategies are crucial to overcome lung protective responses (49). A tantalizing hypothesis is that these stealth strategies were initially devoted to colonize the complex environment of the gastrointestinal tract in an asymptomatic manner, but, when they were deployed to other tissues, such as the lung, they resulted in lethality. Future studies are warranted to validate this hypothesis by comparing the stealth strategies deployed by Klebsiella in the small intestine, the colon, and the lung.

Studies done in mice that were pretreated with antibiotics have revealed bacterial factors that are required for the infection of the gastrointestinal tract (11). These factors then play a role when the colonization resistance is disrupted in immunocompetent mice. Therefore, there is a gap in our understanding of the factors employed by K. pneumoniae to counteract colonization resistance. Here, we uncover that the CPS is necessary for the colonization of the large intestine but dispensable for the colonization of the small intestine, illustrating a hitherto unknown, gastrointestinal, microenvironment-dependent role of the CPS. The fact that the CPS is needed to colonize the large intestine of antibiotic pretreated mice (25, 26) reveals the dual role of this virulence factor in overcoming colonization resistance and in colonizing the gut when the barrier is disrupted. In the case of the former, there is evidence uncovering the role of the CPS as an immunity factor against a T6SS attack (50). Our findings add further weight to the notion that the CPS is the single most important factor governing K. pneumoniae-host interactions *in vivo*. Intriguingly, at the present time, it appears that the CPS is only dispensable in the case of urinary tract infections (51), questioning what makes the urinary tract so different, compared to other tissues, in which the CPS is dispensable.

We also tested the requirement of the T6SS in gastrointestinal colonization. Previous work from our laboratory demonstrated the antimicrobial activity of K. pneumoniae T6SS against bacteria and fungi (27), making it plausible that the T6SS contributes to overcoming the colonization resistance imposed by the microbiome. Indeed, in our model, the T6SS was required to colonize the small and large intestine. Furthermore, our data suggest that the primary role of the T6SS is to outcompete the gut microbiome. Similar results have been reported for other enteric pathogens, such as Shigella sonnei and *S.* Typhimurium (52, 53), illustrating the evolutionarily conserved role of the T6SS to survive in the gut, independently of the infection biology of a pathogenic stealth colonizer, such as K. pneumoniae, or an invasive inflammatory pathogen, such as *Shigella* or Salmonella.

Finally, it is worth discussing the translational opportunities offered by our model. Now, it is possible to conduct functional genomic studies to identify the factors required for K. pneumoniae to overcome colonization resistance and to better understand the differences between metastatic Klebsiella and those strains that do not disseminate to other tissues. This work provides a comprehensive understanding of the interface between K. pneumoniae, the gastrointestinal tract, the innate immune system, and the microbiome. Our model also allows for the investigation of which factors, other infections, or treatments, for example, facilitate colonization or trigger the dissemination of K. pneumoniae from the gut. This knowledge is relevant to the identification of the risks associated with K. pneumoniae invasive infections, which are known to arise from gastrointestinal colonization. Lastly, we envision that our model will be an excellent platform upon which to test therapeutics aiming to eliminate the asymptomatic colonization of K. pneumoniae.

## MATERIALS AND METHODS

### Ethics statement.

The experiments involving mice were approved by the Queen’s University Belfast’s Ethics Committee and were conducted in accordance with the UK Home Office regulations (project licences PPL2778 and PPL2910) issued by the UK Home Office. The infection protocol adhered to the ARRIVE and NC3Rs guidelines. The animals were randomized for interventions, but the researchers who processed the samples and analyzed the data were aware which intervention group corresponded to which cohort of animals.

### Mice.

All experiments were carried out in Queen’s University Biological Services Unit and involved challenging 8 to 9-week-old mice of both sexes. C57BL/6 mice were purchased from Charles River Laboratories and placed in sterile cages upon their arrival and at least 1 week prior to the start of the experimentation for acclimation. The animals were supplied with food and water *ad libitum* and were placed in individually ventilated cages when moved to the Biosafety Level 2 laboratory in which the infection experiments were performed.

### Bacterial strains and growth conditions.

Kp52145 is a K. pneumoniae clinical isolate (serotype O1:K2; sequence type ST66) that has been previously described (13, 54). The capsule mutant strain, 52145-Δ*manC*, and the type VI secretion system mutant strain, 52145-Δ*clpV*, are isogenic strains of Kp52145, and they have been described previously (27, 39). No differences in antibiotic susceptibility were observed between Kp52145 and 52145-Δ*clpV* (Fig. S5). NJST258-1 (serotype O1:K2; sequence type ST258), ATCC 43816 (serotype O1:K2; sequence type ST493), and SGH10 (serotype K1; sequence type ST23) are also K. pneumoniae strains that have been previously described (15–17). K. oxytoca MG2, *K. variicola* MG2, and E. cloacae MG1 were isolated from the small intestines of mice infected with 52145-Δ*clpV* after antibiotic treatment.

10.1128/mbio.03121-22.5 FIG S5 Effect of the antibiotic cocktail on the growth of K. pneumoniae strains. The growth of Kp52145 and the type VI secretion system mutant strain, 52145-Δ*clpV* (Δ*clpV*), in LB without any antibiotic or with the antibiotic cocktail (ampicillin 1g/L, neomycin sulfate 1 g/L, metronidazole 1 g/L, and vancomycin 0.5 g/L). Also, tests using 1:10 and 1:100 dilutions were conducted. For these experiments, 5 μL of overnight cultures were diluted in 250 μL of LB and incubated at 37°C with continuous, normal shaking in a Bioscreen C Automated Microbial Growth Analyzer (MTX Lab Systems, Vienna, VA, USA). The optical density (OD; 600 nm) was measured and recorded every 20 min. Download FIG S5, PDF file, 0.4 MB. Copyright © 2023 Calderon-Gonzalez et al. 2023 Calderon-Gonzalez et al. https://creativecommons.org/licenses/by/4.0/ This content is distributed under the terms of the Creative Commons Attribution 4.0 International license.

All bacteria were grown in 5 mL Luria-Bertani (LB) medium at 37°C on an orbital shaker (180 rpm).

### Gut colonization model.

Overnight bacterial cultures were refreshed 1/10 into a new tube that contained 4.5 mL of fresh LB media. After 2.5 h of incubation on an orbital shaker (180 rpm) at 37°C, the bacteria were pelleted (2,500 × *g*, 20 min, RT) and suspended in PBS. The suspension was adjusted to an OD_600_ of 1.0 to obtain a concentration of 5 × 10^8^ CFU/mL.

At the start of the gut colonization experiments, the animals were administered 200 μL of a 0.2 M solution of sodium bicarbonate via oral gavage. 5 min later, the mice were infected with 100 μL of the desired inoculum. The mice were weighed and checked daily to detect any signs of distress. Animals showing moderate signs of distress (inactivity, separation from the group, hunched posture, and/or body weight loss of >10% over two consecutive days or >20% within 24 h) were euthanized in accordance with approved schedule 1 protocol. Lungs and spleens were plated to assess the dissemination of the bacteria.

### Quantification of the K. pneumoniae burden.

The small intestine, cecum, colon, fecal samples, lungs, liver, and spleen from the mice were weighed, immersed in 1 mL of PBS, and processed for bacterial quantification. In the cases of the gut samples, the intestinal content was previously removed by gently pushing it from one end of the gut and out the other end. Samples were homogenized via mechanical disruption with a sterile plastic loop (fecal samples) or with a Precellys Evolution tissue homogenizer (Bertin Instruments), using 1.4 mm ceramic (zirconium oxide) beads at 4,500 rpm for 7 cycles of 10 s, with a 10 s-pause between each cycle (small intestine, cecum, colon, lungs, liver, and spleen).

Homogenates were serially diluted in sterile PBS and plated onto Salmonella*-Shigella* agar (Sigma-Aldrich) (lungs, liver, and spleen) or SCAI medium (Simmons Citrate [Sigma-Aldrich] agar with 1% inositol) (small intestine, cecum, colon and fecal samples) for the identification of K. pneumoniae colonies. Colonies were enumerated after overnight incubation at 37°C in the cases of Salmonella*-Shigella* agar or after 72 h of incubation in the cases of the SCAI plates. Routinely, and in cases of doubts whether the inositol positive colonies corresponded to the infecting strain, we tested random colonies from the plates for tellurite resistance (via replicate plating on LB agar plates supplemented with 3 μg/mL potassium tellurite; marker for Kp52145), and for the presence of a colibactin island (marker for Kp52145 and SGH10) or the capsule regulator *rmpA* (marker for Kp52145, Kp43816, and SGH10) (via PCR).

The plating detection limits were as follows: 3 CFU/g (small intestine), 7 CFU/gr (large intestine), 20 CFU/gr (cecum), 10 CFU/gr (colon), 10 CFU/gr (lung), 2 CFU/gr (liver), 20 CFU/gr (spleen), and 100 CFU/gr (feces).

### Histology analysis.

The small intestine and colon were excised from the animals, and their contents were removed as previously described. Further cleaning was performed by gently passing them through the lumen 1 mL of 4% paraformaldehyde (PFA). The organs were fixed for 24 h in an excess volume of 4% PFA and were placed in PBS until processing.

For processing, the samples were dehydrated and embedded in paraffin. 10 μm sections were prepared. Hematoxylin and eosin (H&E) staining of the sections was performed, according to standard protocols. Images of the H&E stained slides were obtained using a Eclipse 80i microscope (Nikon) with a 20×/0.8 NA objective, and digital images were acquired using the NIS-Elements software (Nikon).

Blinded evaluation was performed by a researcher of the laboratory using the histopathology scoring indicated in Table S1.

### RNA isolation and RT-qPCR.

Content-cleaned small intestine, cecum, and colon were collected in RNA stabilizing solution and were homogenized with a handheld homogenizer in 1 mL of TRIzol reagent (Ambion) for total RNA extraction, according to the manufacturer’s instructions. The extracted RNA was treated with DNase I (Roche), precipitated with sodium acetate and ethanol, and quantified using a Nanovue Plus spectrophotometer (GE Healthcare Life Sciences). cDNA was generated via the retrotranscription of 1 g of total RNA using M-MLV reverse transcriptase (Invitrogen) and random primers (Invitrogen).

10 nanograms of cDNA were used as a template in a 5 μL reaction mixture from a KAPA SYBR FAST qPCR kit (Kapa Biosystems). The primers used are listed in Table S2. RT-qPCR was performed using a Rotor-Gene Q (Qiagen) with the following thermocycling conditions: 95°C for 3 min for hot-start polymerase activation followed by 40 cycles of 95°C for 5 s and 60°C for 20 s. The fluorescence of SYBR green dye was measured at 510 nm. The relative quantities of mRNAs were obtained using the ΔΔC_T_ method by using hypoxanthine phosphoribosyltransferase 1 (*hprt*) gene normalization.

10.1128/mbio.03121-22.7 TABLE S2 Primers used in this study. Download Table S2, PDF file, 0.10 MB. Copyright © 2023 Calderon-Gonzalez et al. 2023 Calderon-Gonzalez et al. https://creativecommons.org/licenses/by/4.0/ This content is distributed under the terms of the Creative Commons Attribution 4.0 International license.

### Microbiome analysis.

The small intestine and colon fecal contents were collected in sterile tubes and stored at −80°C until processed. Microbial DNA was extracted using a QIAamp PowerFecal Pro DNA Kit (Qiagen), according to the manufacturer’s instructions, using a Precellys Evolution tissue homogenizer (Bertin Instruments) at 5,000 rpm for 2 cycles of 30 s, with a 30 s-pause between them, to homogenize the samples. The extracted DNA was quantified using a Nanovue Plus spectrophotometer (GE Healthcare Life Sciences).

The processing and sequencing of the DNA samples were performed by the Genomics Core Technology Unit of Queen’s University Belfast. Libraries were constructed in batches, quantified and run on an Illumina Miseq V2 Platform with a read length of 500 bp (2 × 250 bp) and a read depth of 100.000 reads/sample. 16S amplicon PCR of the V3-V4 region was performed using the 341F (CCTACGGGNGGCWGCAG) and 806R (GGACTACHVGGGTWTCTAAT) primers.

Amplicon identification, quantification, and analyses were carried out in R 4.1.1, using different packages. First, a quality assessment of the sequences was performed using FASTQC software (https://www.bioinformatics.babraham.ac.uk/projects/fastqc/), and then a DADA2 (55) version 1.24 pipeline was used to generate the amplicon sequence variants (ASVs). In brief, it consisted of a first stage in which sequences were inspected visually prior to the application of the filterAndTrim function (trimming 13 bases from each sequence, removing sequences containing Ns, and applying a filter for the Expected Errors [maxEE] of 2 and 6 for each of the reads), retaining 76% of the reads, on average. Then, error models were learned and applied to denoise and merge the reads, using default the parameters, prior to the removal of chimeric sequences. The SILVA v138 database was used for the taxonomic assignment, using a minimum bootstrap confidence of 80. A total of 2,988 ASVs were identified, transformed into a phyloseq object for further analyses, and further filtered down via the removal of sequences assigned to mitochondria as well as those without a phylum or class. Further filtering based on prevalence and abundance resulted in a total of 819 ASVs. The alpha and beta diversities were analyzed using Phyloseq v1.40 (56), and a differential abundance analysis was carried out with Deseq2 v1.36.0, using a statistical significance threshold of an adj. *P* value of 0.05.

### Postinfection antibiotic treatment.

C57BL/6 mice were infected with 1 × 10^7^ CFU of the ampicillin-resistant K. pneumoniae strain ATCC 43186, as described before. On day 6 and 9 postinoculation, they received 500 mg/kg of ampicillin sodium salt (Sigma-Aldrich) or vehicle (sterile water) intraperitoneally. 24 h after the second dose, the animals were euthanized, and their organs were collected for bacterial burden quantification, as previously described.

### Microbiota depletion.

C57BL/6 mice were treated for 10 days with a mix of broad-spectrum antibiotics in their drinking water (ampicillin 1g/L, neomycin sulfate 1 g/L, metronidazole 1 g/L, and vancomycin 0.5 g/L) (Sigma-Aldrich) (Brown et al., 2017). Antibiotic therapy was stopped 3 days prior to infection, and it was replaced with normal, antibiotic-free drinking water. The mice were infected with 1 × 10^7^ CFU of either Kp52145 or 52145-Δ*clpV* strains via oral gavage, as previously described. The animals were euthanized 72 h postinoculation, and their organs were processed for bacterial quantification, as previously described.

### Identification of bacterial colonies via MALDI.

When required, the identification of bacterial colonies was performed using a Vitek MS instrument (bioMérieux, France) in accordance with the manufacturer’s instructions. Briefly, fresh cultures were prepared on appropriate media, from which a single bacterial colony or a part of a colony was transferred to the target slide as a smear. Then, 1 μL of matrix (Vitek MS-CHCA) was added and air dried. Following the insertion of the loaded slide into the Vitek MS, operating with software version 1.6.0, spectra were generated from the bacterial suspensions by the instrument, and the generated spectra were compared to reference spectra in the database to provide an identification. Each identification was reported with a score, expressed as a percentage, indicating the degree of confidence in that result. Scores of >90% were considered to be decisive.

### Detection of K. pneumoniae by ZKIR-qPCR.

The DNA samples used for the microbiome analysis were analyzed via the ZIKIR-qPCR method, which has previously been described in the detection of the presence of K. pneumoniae (23, 24). DNA was amplified in a 5 μL PCR mix that contained 0.4 μL of each ZKIR primer (final concentration, 200 nM each) (Table S2), 2.6 μL of KAPA SYBR FAST qPCR mix, and 2 μL of sample DNA. A standard curve was also generated using serial dilutions of genomic DNA that were extracted from Kp52145 under the same conditions, with the number of genome copies per dilution being calculated according to the following equation:

$$\mathrm{genome}\;\mathrm{copy}\;\mathrm{number}\;=\;[\left(\mathrm{mass}\;\mathrm{of}\;\mathrm{input}\;\mathrm{DNA}\;\mathrm{in}\;\mathrm{ng}\right)\;*\;\left(6.0221*10^{23}\;m\mathrm{olecules}/\mathrm{mole}\right)]/\left(\mathrm{length}\;\mathrm{of}\;\mathrm{genome}\;\mathrm{in}\;\mathrm{bp}\;*\;660\;g/\mathrm{mol}\;*\;10^{9}ng/g\right)$$

where the length of the K. pneumoniae genome is 5.5 × 10^6^ bp.

RT-qPCR was performed using a Rotor-Gene Q (Qiagen) with the following thermocycling conditions: 95°C for 3 min for hot-start polymerase activation followed by 40 cycles of 95°C for 5 s and 60°C for 20 s.

### Antibacterial competition assay.

Attacker K. pneumoniae strains and preys were grown until the mid-exponential phase, collected via centrifugation, and resuspended in PBS to an OD_600_ of 1.2. The attacker and preys were mixed at a 10:1 ratio by volume, and 100 μL of the mixture were spotted in a prewarmed agar plate at 37°C. The contact spot was incubated for 6 h. Recovered cells were plated out on antibiotic selective media, and viable cells were reported as recovery target cells, representing the CFU per mL of the recovered prey after coculture. *K. variicola*, K. oxytoca, and E. cloacae were selected via plating on 25 μg/mL carbenicillin, and 52145-Δ*clpV* was selected via plating on LB agar containing 3 μg/mL sodium tellurite. All experiments were carried out with triplicate samples on at least three independent occasions.

### Construction of *clpV* mutants.

Strains were grown in Luria-Bertani media, supplemented with appropriate antibiotics at the following concentrations: gentamicin (30 μg/mL), chloramphenicol (30 μg/mL), kanamycin (100 μg/mL), and diaminopimelic acid (DAP, 300 μM). Bacterial genomic DNA was extracted using a PureLink Genomic DNA Kit (Life Technologies), as per the manufacturer’s instructions. DNA fragments targeting the *clpV* gene were amplified from genomic DNA using KAPA HiFi DNA polymerase (Roche). The primers were designed from conserved regions identified via the comparison of the *clpV* gene with the the available *K. variicola* and K. oxytoca genomes on NCBI GenBank (accessed 01/07/22 and 04/02/2022, respectively). Primers are shown in Table S2.

To construct a K. oxytoca *clpV* mutant, an internal fragment of *clpV* was amplified via PCR, using primers pKNOCK-Koxy-clpV-F3 and pKNOCK-Koxy-clpV-R3. The PCR product was gel purified, cloned into the pKNOCK-cm suicide vector (57), and cut with BamHI (New England Biolabs) via homologous alignment cloning (58) to generate pKNOCKClpV_oxy_. This plasmid was transformed into the E. coli DAP auxotroph β2163 (59), which then mobilized the plasmid to K. oxytoca MG2. A mutant in which pKNOCKClpV_oxy_ was inserted into the *clpV* locus via homologous recombination was confirmed via PCR using primers pKNOCK-Koxy-clpV-sc-F and pKNOCK-Koxy-clpV-sc-R, and it was named K. oxytoca*-ΔclpV*.

To construct a *K. variicola clpV* mutant, we used λ-red-mediated homologous recombination, as described previously (60). Homologous regions upstream and downstream of *clpV* were amplified via PCR, using primers 6144-clpV.2 and 6144-clpV.3 as well as 6144-clpV.4 and 6144-clpV.5, respectively, and they were sewn together with the kanamycin cassette amplified from pKD4 (61), using primers cm.3a and cm.4a. KAPA HiFi was used as the polymerase. The PCR products were amplified with using primers 6144-clpV.2 and 6144-clpV.5, and the PCR fragment was purified with a Qiagen MinElute Kit. Strain *K. variicola* MG2 was transformed with pKOBEG-gent, which encodes the λ phage redγβα operon (62) and was expressed under the control of the arabinose-inducible pBAD promoter. Briefly, *K. variicola* were grown overnight in LB and were then subcultured 1:100 in 200 mL of LB supplemented with gentamicin and 0.2% arabinose. Cells were grown at 37°C until reaching an OD_600_ of 0.4. The cells were cooled on ice for 30 min, pelleted, resuspended in ice-cold 10% glycerol, and incubated on ice for 1 h. The cells were washed three times with ice-cold 10% glycerol and were finally suspended in 200 μL of 10% glycerol. The cells were electroporated with 1 μg 3-way-PCR DNA in a 2 mm cuvette with 2.5 kV, 200 Ω, and 25 μF. The cells were recovered in 1 mL of SOC media (2% tryptone, 0.5% yeast extract, 10 mM NaCl, 2.5 mM KCl, 10 mM MgCl_2_, 10 mM MgSO_4_, and 20 mM glucose) at 37 C for 2 h. Transformed cells were selected via plating on LB agar supplemented with kanamycin. The replacement of the wild-type allele by the mutant was confirmed via PCR using primers 6144-clpV.1 and 6144-clpV.6, and the mutant was named *K. variicola-ΔclpV.* The pKOBEG-gent plasmid was cured from the mutant strain via overnight growth at 37°C.

### Quantification and statistical analysis.

Statistical analyses were performed using a one-way analysis of variance (ANOVA) with the Bonferroni correction, a two-tailed *t* test, or, when the requirements were not met, a Mann-Whitney U test. *P* values of <0.05 were considered to be indicative of a statistically significant result. The normality and equal variance assumptions were tested using the Kolmogorov-Smirnov test and the Brown-Forsythe test, respectively. All analyses were performed using the GraphPad Prism for Windows (version 9.4.1) software package.

### Data availability.

The data are available at NCBI SRA (bioproject PRJNA880484).
