# Supplementary material for: Modelling the Gastrointestinal Carriage of Klebsiella pneumoniae Infections
Source: mBio. 2023 Jan 4;14(1):e03121-22. doi: 10.1128/mbio.03121-22 (PMC9972987; doi:10.1128/mbio.03121-22)
Supplement: FIG S1 [file mbio.03121-22-s0001.pdf]

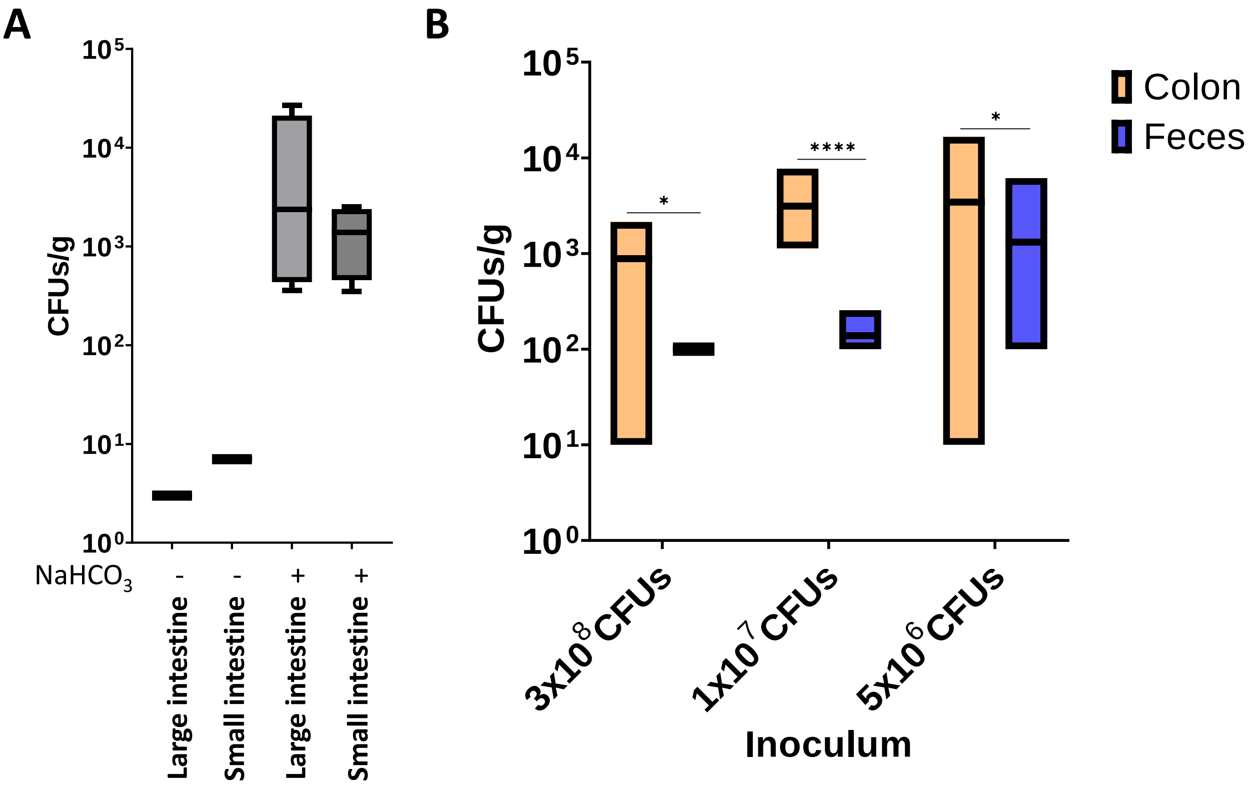

Figure S1. Sodium bicarbonate treatment facilitates *K. pneumoniae* gut colonisation.

A. CFUs per gr of small intestine and colon of mice infected with 10^8 CFUs of Kp52145. Mice were pre-treated or not with sodium bicarbonate 5 min before oral gavage of bacteria. 4-5 mice per group were infected. **B.** Bacterial loads in the colon and faeces of mice infected with the indicated bacterial doses. Samples were obtained twelve days post infection. 4-5 mice per group were analysed.

In panels, values are presented as the mean ± SD. ****P ≤ 0.0001; *P ≤ 0.05 for the indicated comparisons determined using Mann-Whitney U test.
