# Supplementary material for: Modelling the Gastrointestinal Carriage of Klebsiella pneumoniae Infections
Source: mBio. 2023 Jan 4;14(1):e03121-22. doi: 10.1128/mbio.03121-22 (PMC9972987; doi:10.1128/mbio.03121-22)
Supplement: TABLE S1 [file mbio.03121-22-s0006.pdf]

Table S1. Histopathology scoring for murine intestine.

Score	Infiltration of inflammatory cells	Submucosal edema	Epithelial damage	Exudate
3	Severe multifocal to diffuse infiltration	Severe (>40%)	Multifocal to diffuse ulceration and/or severe multifocal to diffuse enterocyte hyperplasia with metaplasia [colon]<br><br>Severe multifocal to diffuse villus blunting associated with multifocal to diffuse ulceration and/or haemorrhage [small intestine]	Severe accumulation
2	Moderate multifocal to diffuse infiltration	Moderate (20-40%)	Moderate multifocal to diffuse loss of goblet cells associated with enterocyte hyperplasia [colon]<br><br>Moderate multifocal villus blunting associated with mild to moderate epithelial erosion [small intestine]	Moderate accumulation
1	Mild multifocal to diffuse infiltration	Mild (1-20%)	Erosion/mild loss of goblet cells and enterocyte hyperplasia [colon]<br><br>Mild multifocal villus blunting [small intestine]	Mild accumulation
0	No infiltration	Not present	Not present	Not present
