# Supplementary material for: Modelling the Gastrointestinal Carriage of Klebsiella pneumoniae Infections
Source: mBio. 2023 Jan 4;14(1):e03121-22. doi: 10.1128/mbio.03121-22 (PMC9972987; doi:10.1128/mbio.03121-22)
Supplement: FIG S2 [file mbio.03121-22-s0002.pdf]

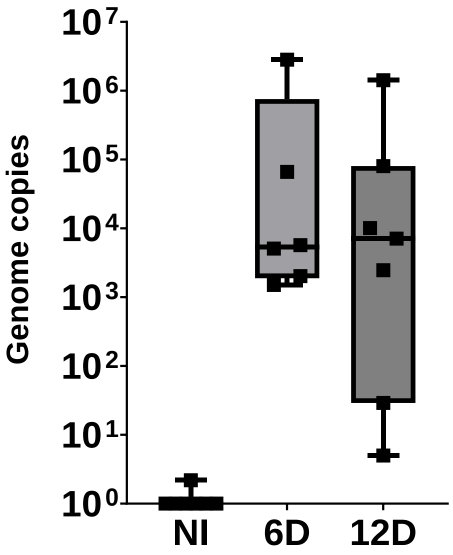

Figure S2. Detection of *K. pneumoniae* by ZKIR-qPCR in colon samples.

K. pneumoniae was detected in the colon samples of non-infected (NI) mice infected and of infected mice at six (6D) and twelve (12D) days post infections by the ZKIR-qPCR method. 6-7 mice were analysed in each group.
