# Supplementary material for: Modelling the Gastrointestinal Carriage of Klebsiella pneumoniae Infections
Source: mBio. 2023 Jan 4;14(1):e03121-22. doi: 10.1128/mbio.03121-22 (PMC9972987; doi:10.1128/mbio.03121-22)
Supplement: FIG S3 [file mbio.03121-22-s0003.pdf]

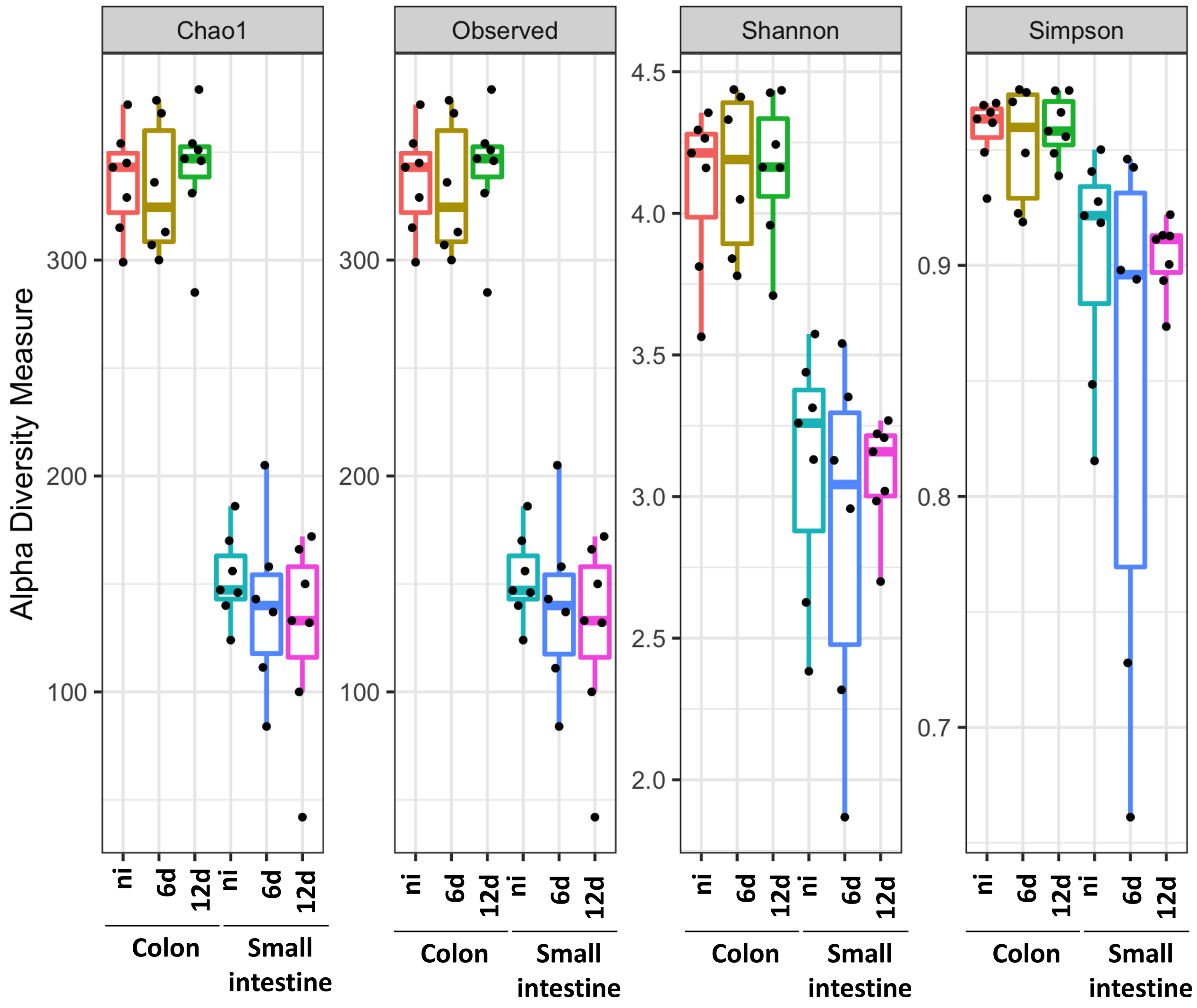

Figure S3. ***K. pneumoniae*** **gut colonisation does not affect the richness of the microbiome.**

Diversities of the faecal microbiota of non-infected (ni) mice and of infected mice at six (6d) and twelve (12d) days post infections were summarized by four indexes. 6-7 mice were analysed in each group. No statistical differences were found.
