# Supplementary material for: Modelling the Gastrointestinal Carriage of Klebsiella pneumoniae Infections
Source: mBio. 2023 Jan 4;14(1):e03121-22. doi: 10.1128/mbio.03121-22 (PMC9972987; doi:10.1128/mbio.03121-22)
Supplement: FIG S4 [file mbio.03121-22-s0004.pdf]

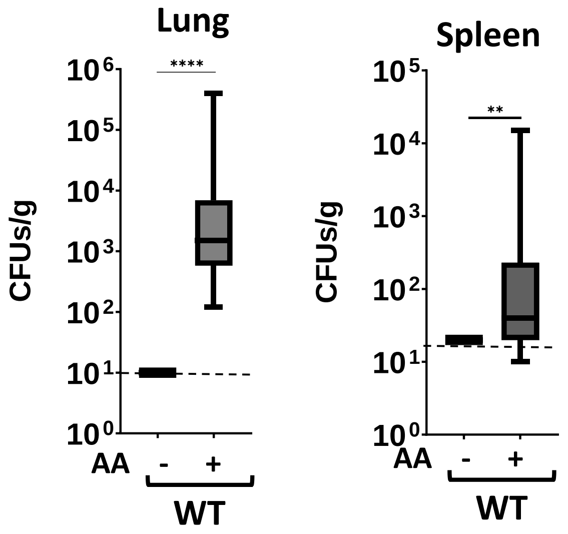

Figure S4. ***K. pneumoniae*** **disseminates from the gut to other tissues in mice pre-treated with antibiotic cocktail.**

CFUs per gr of lung and spleen of mice infected with Kp52145 which were pre-treated or not with the antibiotic cocktail (AA). 10-16 mice were included in each group in two independent experiments.

Dashed lines indicate the limit of detection. In all panels, values are presented as the mean ± SD. ****P ≤ 0.0001; ** P ≤ 0.01 for the indicated comparisons determined using Mann-Whitney U test.
