# Supplementary material for: Modelling the Gastrointestinal Carriage of Klebsiella pneumoniae Infections
Source: mBio. 2023 Jan 4;14(1):e03121-22. doi: 10.1128/mbio.03121-22 (PMC9972987; doi:10.1128/mbio.03121-22)
Supplement: FIG S5 [file mbio.03121-22-s0005.pdf]

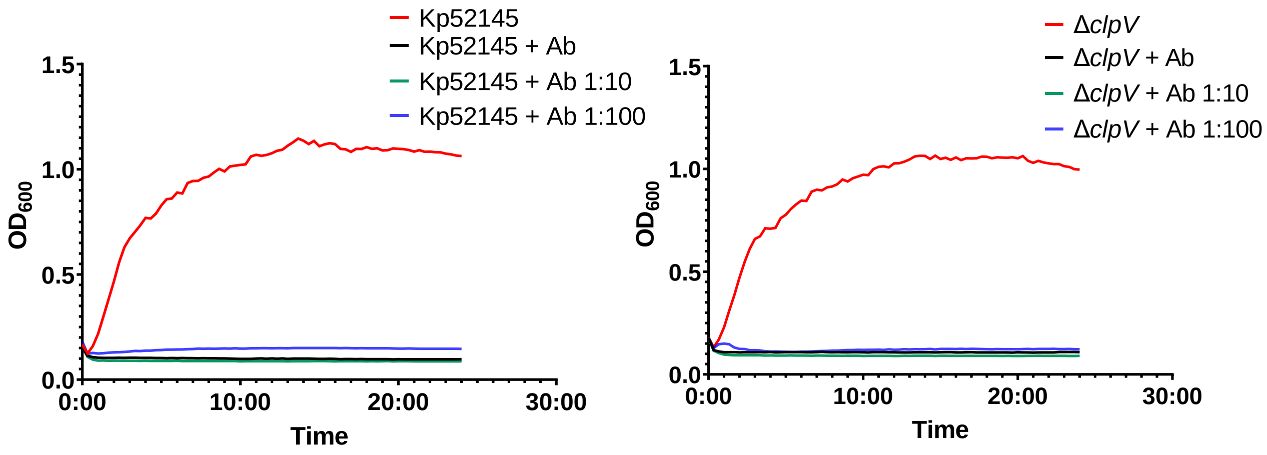

Figure S5. Effect of the antibiotic cocktail on the growth of *K. pneumoniae* strains.

The growth of Kp52145 and type VI secretion system mutant strain, 52145-Δ*clpV* (Δ*clpV*), in LB without any antibiotic or with the antibiotic cocktail (ampicillin 1g/l, neomycin sulfate 1 g/l, metronidazole 1 g/l, and vancomycin 0.5 g/l) tested also ten (1:10) and one hundred (1:100) times diluted was determined.

For these experiments, 5 µl of overnight cultures were diluted in 250 µl of LB and incubated at 37°C with continuous, normal shaking in a Bioscreen C Automated Microbial Growth Analyzer (MTX Lab Systems, Vienna, VA, USA). Optical density (OD; 600 nm) was measured and recorded every 20 min.
