# Supplementary material for: Modelling the Gastrointestinal Carriage of Klebsiella pneumoniae Infections
Source: mBio. 2023 Jan 4;14(1):e03121-22. doi: 10.1128/mbio.03121-22 (PMC9972987; doi:10.1128/mbio.03121-22)
Supplement: TABLE S2 [file mbio.03121-22-s0007.pdf]

Table S2. Primers used in this study.

Name	Sequence (5’-3’)
**qPCR**	
m_Cxcl1_F1	TGC ACC CAA ACC GAA GTC ATA G
m_Cxcl1_R1	TTG TAT AGT GTT GTC AGA AGC CAG C
mHPRT-F1	GAT CAG TCA ACG GGG GAC AT
mHPRT-R1	GGT CCT TTT CAC CAG CAA GC
mReg3g_for	ATG GCT CCT ATT GCT ATG CC
mReg3g_rev	GAT GTC CTG AGG GCC TCT T
Reg4F_qPCR	CGC TGA GAT GAA CCC CAA G
Reg4R_qPCR	TGA GAG GGA AGT GGG AAG AG
S100a8F_qPCR	AGT GTC CTC AGT TTG TGC AG
S100a8R_qPCR	ACT CCT TGT GGC TGT CTT TG
m_Tnf_F1	GAT CGG TCC CCA AAG GGA TG
m_Tnf_R1	CAC TTG GTG GTT TGC TAC GAC
**Construct *K. oxytoca clpV* mutant**	
pKNOCK-Koxy-clpV-F3	TAGAACTAGTggatcc ATGAGTGGGATATGGAGATTC
pKNOCK-Koxy-clpV-R3	GCAGCCCGGGggatcc ATCTACCGCCTTGTCAGG
**Check *K. oxytoca clpV***	
pKNOCK-Koxy-clpV-sc-F	CGAGATTACTATTGAGCACTGG
pKNOCK-Koxy-clpV-sc-R	GGCTCAGTACAGGCAGAATC
**Construct *K. variicola clpV* mutant**	
6144-clpV.1	CCAGACTCCGGTTATCATGC
6144-clpV.2	ATTTGCCTAAGCCATTCAACG
6144-clpV.3	ggaataggaactaaggaggaCAAACTTCCAATCATGAAGC
6144-clpV.4	cctacacaatcgctcaagacCTGGGTGGTCTGCGTTTAAC

6144-clpV.5	CGAAAACCTCTCCACCACC
6144-clpV.6	GCGCGCTGTGTCATTATCC
cm.3a	TCCTCCTTAGTTCCTATTCC
cm.4a	GTCTTGAGCGATTGTGTAGG
**ZIKIR primers**	
ZKIR_F	CTAAAACCGCCATGTCCGATTTAA
ZKIR_R	TTCCGAAAATGAGACACTTCAGA
